# Some Applications of Nanobiotechnology in Parasitology

**Published:** 2019-09

**Authors:** Kareem HATAM-NAHAVANDI

**Affiliations:** Department of Microbiology and Immunology, School of Medicine, Iranshahr University of Medical Sciences, Iranshahr, Iran

## Dear Editor-in-Chief

Parasitic diseases are associated with major morbidity and mortality worldwide (1). Therefore, the best strategy adopted to tackle the aforementioned crisis associated with parasitic diseases is to develop new biotechnologies in order to improve the efficacy and specificity of diagnostic tests, and the tolerability of existing antiparasitic agents. In this review, we would discuss some nanobiotechnology applications in the diagnosis and treatment of parasitic diseases.

Before clinicians can detect a parasitic disease, diagnostic methods focus on the visible symptoms of patients in most parasitic diseases. However, treatment may show reduced efficacy by the time the symptoms appear; as a result, earlier detection is associated with a greater chance of treatment. The ideal situation involves diagnosis and treatment of parasitic diseases before manifestation of symptoms. Nucleic acid diagnostics play an important role, as parasitic diseases can be detected at an early symptomless stage, which is more responsive to effective treatment.

In this regard, nanobiotechnology by integrating semiconductor nanocrystals or quantum dots (QDs) presents a solution. In comparison with typical organic molecules more readily decomposed, the minuscule probes in nanobiotechnology can withstand a considerably larger number of excitation cycles and light emission (2). In different bioimaging applications and in vitro diagnostics, QDs have been recently applied owing to their large stoke shifts, high photostability, and tunable narrow-emission spectral features. These fluorescent properties of QDs allow their application as a robust fluorophore to label microorganisms, including intracellular organelles, red blood cells (RBCs), genes, and proteins. QDs can be also used as a probe for anti-malarial drug screening (2).

The antibody-conjugated QDs can readily and specifically identify parasites, including *Cryptosporidium parvum* and *Giardia duodenalis* through antibody-antigen interaction and recognition. For these immunoassays, different colored QDs, which act as immunoassay labels, are conjugated to organism-specific antibodies subsequently detected. QDs in immunofluorescent labeling of *C. parvum* oocysts present more consistent and important results in water samples (2).

Medical communities insist on using colorimetric and optical methods, regardless of the benefits of magnetic imaging. Nanosphere (Northbrook, Illinois) is an organization, proposing techniques for optical detection of the genetic composition of biological specimens. For any genetic sequence, gold nanoparticles (NPs) with short DNA segments facilitate the easy-to-read test. If the target sequence is present in the sample, it can bind to complementary DNA tentacles on multiple nanospheres and create a dense web of visible gold balls.

Verigene Enteric Pathogens Flex Test (EP Flex) (VERIGENE® gastrointestinal infection tests) is the first comprehensive sample-to-result test that will detect eight of the most common intestinal parasites directly from stool specimens in Cary-Blair and non-formalin-based fixatives (e.g. Total-Fix, EcoFix). In addition to the intestinal protozoa detection, EP Flex will also simultaneously detect eight bacterial enteric pathogens, five viral enteric pathogens, and four toxin-mediated enteric pathogens. The test is performed on Nanosphere’s Verigene Flex System, a new sample-to-result, high-throughput platform that leverages Nanosphere’s gold NP chemistry in a user-friendly format (3).

For determining the biological phenotype in healthy and unhealthy states, proteins play a major role and represent functionality. Accordingly, proteomics is of great significance in diagnostics and pharmaceuticals, where the signaling pathways may alter by drugs. Small modular protein components or chemical groups can also be used to treat protein chips; they can bind to proteins with a certain biochemical or structural motif. In recent years, chemical proteomics has been developed as a powerful complementary strategy for drug-target discovery. This approach uses small drug-like molecules that can be bound to a ligand immobilized on a solid support or exposed to protein chips. Subsequently, those proteins bound to the ligand are identified as potential drug targets.

Proteomic analyses tend to highlight the most abundant proteins in a sample at the expense of less abundant, but not less important proteins that may also contribute to a specific phenotype. Therapeutic NPs can be delivered to targets, including sites which are not simply accessible to standard drugs. When a therapeutic agent attaches to NPs chemically, it can move towards the disease or infection site through magnetic or radio signals. In addition, these drugs may be developed in a way to release only when there are specific molecules. Moreover, by decreasing the effective concentration for treatment, we can prevent the negative side effects of potent medications. In addition, release can be managed more accurately through drug encapsulation in NPs. Today, by using nanotechnology, different agents, which cannot be orally administered owing to their poor bioavailability, can be applied in treatment processes (4).

Liposomes are spherical vesicular structures composed of phospholipid bilayers, concentrically oriented around an aqueous compartment, serving as a carrier of hydrophilic or lipophilic drugs. In fact, by improving drug absorption, decreasing drug toxicity, and increasing biological half-life, the therapeutic effects of encapsulated drugs improve. These structures are both bio-compatible and biodegradable and interact with cells through endocytosis, simple adsorption, lipid exchange, and fusion with cell membranes. They have been used to deliver artemisinin and its derivatives with proper biological activity, compared to free artemisinin and its derivatives. For antileishmanial therapy, drug targeting can be achieved by liposomal encapsulated drugs, allowing them to reach intracellular *Leishmania* amastigotes (5). The drug-containing liposomes naturally enter the macrophages by phagocytosis, and hence, the drug is delivered passively to the phagolysosome where it can directly act on parasites.

In the future, nanomedicine can have great contributions to the treatment and diagnosis of parasitic diseases.

## References

[1] Date AA Joshi MD Patravale VB (2007). Parasitic diseases: Liposomes and polymeric nanoparticles versus lipid nanoparticles. Adv Drug Deliv Rev, 59(6):505–21. 17574295 10.1016/j.addr.2007.04.009

[2] Destura RV Cena RB Galarion JH (2015). Advancing *Cryptosporidium* Diagnostics from Bench to Bedside. Curr Trop Med Rep, 2:150–160.

[3] Fakruddin M. Hossain Z. Afroz H (2012). Prospects and applications of nanobiotechnology: a medical perspective. J Nanobiotechnology, 10:31. 22817658 10.1186/1477-3155-10-31 PMC3422163

[4] Rizvi SAA Saleh AM (2018). Applications of nanoparticle systems in drug delivery technology. Saudi Pharm J, 26(1):64–70. 29379334 10.1016/j.jsps.2017.10.012 PMC5783816

[5] Bruni N Stella B Giraudo L (2015). Nanostructured delivery systems with improved leishmanicidal activity: a critical review. Int J Nanomedicine, 12:5289–5311. 10.2147/IJN.S140363 PMC5536235 28794624

